# Epidemic Impetigo Contagiosa

**Published:** 1903-11

**Authors:** 


### Epidemic Impetigo Contagiosa.

SOME of the most prominent teachers in the school of Vienna are still insisting, in spite of modern revelation, that impetigo contagiosa is merely one of the many varieties of eczema. A volume recently issued under their auspices furnishes illustrations of what they consider typical cases corroborating their contention. This teaching is damaging to those American students who seem to regard a foreign education as superior to that which can be obtained at home, and who return here to disseminate the heresies of the Vienna school.

Impetigo contagiosa must be regarded as a disease *sui generis,* although the bacteriologic findings differ little from those relating to pus otherwise existing. Some investigators claim that the exciting agent is the streptococcus, while others consider it to be a specific coccus, differing from both the conventional staphylococcus aureus and the streptococcus of Fehleisen. At the time of the writer's first acquaintance with the disease, a few years ago, impetigo was represented by the "stuck-on crust" variety of Tilbury Fox, being, however, almost wholly confined to poor children and rarely invading the adult. Since 1898, several phases of the disease have appeared which are more or less worthy of note, especially considering the fact that, up to the present time, our medical magazines and textbooks have published so few articles relating to the subject. The disease no longer confines itself to the poor, or to children, but indiscriminately attacks persons of all classes and ages. It has been epidemic in various portions of Buffalo, and has visited almost every hamlet in Western New York. In the majority of cases, the recognised source of infection was the barbershop. In one little town of four hundred inhabitants, where there was one barber, almost every customer was infected, including those who merely had the hair cut or the neck shaved. The variety was bullous. It would often heal in the center and leave rings, and would extend peripherally by undermining the epidermis with fluid. Although the essential lesion always began as a vesicle, it very rapidly enlarged to from one-quarter to three inches, the augmented lesion appearing as if the skin had been touched with carbolic acid. It was surprising how often the affection was taken for ringworm; in fact, in the majority of cases, it was diagnosticated as such. To this form of impetigo has been applied the popular term "barber's itch," which is a synonym for tenæ baræ, and which should not be mistaken for the circinate form.

Impetigo has a clinical entity, but, nevertheless, it is subject to continual changes, probably due to the infecting organism itself, although climate, humidity and temperature have some influence on these transformations. These facts have contributed more or less to the existing confusion of names by which the average physician is so frequently puzzled. It should be the mission of the medical profession to inform the public regarding the nature of this disease and to interest the barber in all matters relating to it, especially sanitation, which is indispensable. As a rule, the barber is an obstinate proposition, for whenever he is informed of the presence of impetigo in his shop, in the majority of the cases he claims that it is caused by the immorality of his customer, a screen which possesses very wide meshes. The offender can only be reached by iron-clad rules and regulations; and it should be a matter of general interest that many boards of health, including that of Buffalo, have inaugurated measures to enforce observance of existing laws. Besides, the state has appointed three inspectors of barbershops, selected from the profession itself. Would it not have been better if one of these had been a trained man, competent to deal with matters which require medical skill and experience?

The careful consideration of impetigo contagiosa just at this time is important, for within the past few months epidemics of the disease considered here have appeared in Buffalo.

